# The Influence of Multimorbidity on Clinical Progression of Dementia in a Population-Based Cohort

**DOI:** 10.1371/journal.pone.0084014

**Published:** 2013-12-30

**Authors:** René J. F. Melis, Alessandra Marengoni, Debora Rizzuto, Steven Teerenstra, Miia Kivipelto, Sara B. Angleman, Laura Fratiglioni

**Affiliations:** 1 Department of Geriatric Medicine/Radboud Alzheimer Centre, Radboud University Medical Centre, Nijmegen, The Netherlands; 2 Department of Medical and Surgery Sciences, University of Brescia, Brescia, Italy; 3 Aging Research Center/Department of Neurobiology, Care Sciences and Society, Karolinska Institutet and Stockholm University, Stockholm, Sweden; 4 Department of Health Evidence, Radboud University Medical Centre, Nijmegen, The Netherlands; University of California, San Francisco, United States of America

## Abstract

**Introduction:**

Co-occurrence with other chronic diseases may influence the progression of dementia, especially in case of multiple chronic diseases. We aimed to verify whether multimorbidity influenced cognitive and daily functioning during nine years after dementia diagnosis compared with the influence in persons without dementia.

**Methods:**

In the Kungsholmen Project, a population-based cohort study, we followed 310 persons with incident dementia longitudinally. We compared their trajectories with those of 679 persons without dementia. Progression was studied for cognition and activities of daily life (ADLs), measured by MMSE and Katz Index respectively. The effect of multimorbidity and its interaction with dementia status was studied using individual growth models.

**Results:**

The mean (SD) follow-up time was 4.7 (2.3) years. As expected, dementia related to both the decline in cognitive and daily functioning. Irrespective of dementia status, persons with more diseases had significantly worse baseline daily functioning. In dementia patients having more diseases also related to a significantly faster decline in daily functioning. Due to the combination of lower functioning in ADLs at baseline and faster decline, dementia patients with multimorbidity were about one to two years ahead of the decline of dementia patients without any co-morbidity. In persons without dementia, no significant decline in ADLs over time was present, nor was multimorbidity related to the decline rate. Cognitive decline measured with MMSE remained unrelated to the number of diseases present at baseline.

**Conclusion:**

Multimorbidity was related to baseline daily function in both persons with and without dementia, and with accelerated decline in people with dementia but not in non-demented individuals. No relationship of multimorbidity with cognitive functioning was established. These findings imply a strong interconnection between physical and mental health, where the greatest disablement occurs when both somatic and mental disorders are present.

## Introduction

In absence of disease modifying treatment, slowing down the progression of dementia by lessening the cognitive and functional decline is a possible strategy to limit the burden of the dementing disorders [1]. An increasing body of evidence suggests an interrelatedness between physical and cognitive functioning [2]–[4]. Clinical experience suggests that dementia patients with physical co-morbidity progress faster than patients without co-morbidity. This relation may offer a “window” for slowing down dementia progression.

Unfortunately, the relationship between dementia progression and multimorbidity is not frequently studied in the literature. The results of cross-sectional studies have shown contradictory results [5]–[9]. There are only a few longitudinal studies concerning the effect of multimorbidity on dementia progression, which also provided contradictory results with respect to the influence of multimorbidity on dementia progression [10]–[13]. The severity and the progression of dementia are not one-dimensional constructs: the clinical presentation of a dementia at a certain point in time is the result of the functioning on several domains. Global cognitive and daily functioning are core to the diagnosis of dementia and also core outcomes in dementia severity measures such as the Clinical Dementia Rating Scale [14], [15]. Taking this into account, in the current study we studied the effect of multimorbidity on both cognitive and daily functioning in dementia. It is likely that multimorbidity may also affect daily functioning independently of the presence of dementia [16], and such an effect may also be present for cognitive functioning [17]. Therefore, in addition to studying the effect of the presence of multimorbidity on functioning in dementia patients, we were interested to understand if multimorbidity affected progression in daily and cognitive functioning differently in persons with and without a dementia.

With this study we aimed to explore the role of multimorbidity, defined as the presence of two or more chronic diseases, on the change in cognitive and daily function over up to nine years of follow up in a large population-based sample of persons with incident dementia and to compare these findings with the effects in non-demented persons.

## Methods

The study population was recruited among all 2368 inhabitants in the Kungsholmen Parish of Stockholm, Sweden who were 75 years and older at 31 december 1987 (born in or before 1912) [18], of whom 1810 persons agreed to participate in the Kungsholmen Project (KP). Eligible for the current study were participants who were free from dementia at the Kungsholmen Project baseline assessment (n = 1475) and who participated in the first KP follow up assessment (n = 989). The baseline assessment (BL) was carried out between 1987 and 1989 and was followed by four examinations spaced approximately three years apart (FU1-4).

### Ethics Statement and Data Sharing Statement

The Kungsholmen Project has been approved by the Ethics Committee of the Karolinska Institute. The data from the Kungsholmen Project are available upon request.

Of the 989 persons who participated in the first follow up, 310 people were diagnosed with dementia at first (n = 127), second (n = 112), or third follow up (n = 71). The progression of dementia over time was studied by using data from the last follow up assessment in the Kungsholmen Project before dementia onset and onwards, until either a participant died, withdrew informed consent, or reached the third follow up. In order to be included in this study, at least two assessments (one before dementia onset and at least one after dementia onset) had to be available.

The current study enrolled participants from the last clinical assessment carried out in the Kungsholmen Project before a subject developed dementia, because several demented participants had already a huge drop in dementia-related outcome measures over the time frame in which the dementia occurred. It is likely that the observed drop was at least to some extent part of the post-onset trajectory.

In order to compare the trajectories of the incident dementia cases with the trajectories of non-demented persons, we constructed control trajectories for the 679 participants who participated in the first follow up and remained dementia-free throughout the follow up. As dementia onset is a random event, the construction of the trajectories of the non-demented persons was done by randomly assigning one of the assessments they participated in (BL-FU2) as the starting point of their follow up trajectories in this study.

#### Data collection

At all examinations in the Kungsholmen Project the data were collected following the same standardised protocols [18], [19], with the exception of the measurement of disability using the Katz Index [20]. At BL in the Kungsholmen Project the participants were asked several questions about their functioning with regards to the six activities of daily living (ADL) that the Katz Index assesses, and afterwards this information was recoded into three levels of functioning for each of the six abilities (requiring no help, some help, or much help). At the follow ups the participants were directly asked to rate their functioning in each of the six domains on a three point scale (0,1 or 2). This approach resulted in an ADL score which ranges from 0 (‘no help’ in all six domains) to 12 (‘much help’ in all six domains). If the participant was not able to answer the questions reliably, an informant was contacted.

#### Dementia diagnosis

Diagnosis of dementia was based on DSM-III-R-criteria and was defined as memory impairment accompanied by impairment in abstract thinking, judgment, other higher cortical function or personality change, where the resulting cognitive disturbance interfered with work, social activities or relationship with others and without these cognitive changes occurring exclusively in the setting of delirium [14]. If memory impairment was evident but dysfunction of a second cognitive ability was questionable, the additional category ‘questionable dementia’ was used [21]. These persons were regarded as non-demented in this study.

#### Multimorbidity

Multimorbidity was defined as any co-occurrence of two or more chronic conditions in the same individual [22]. The number of chronic conditions at the assessment from which the participants were enrolled in the current study was counted in the same manner as in our previous studies on multimorbidity [23], except for some modifications. We only used the information from the computerized Stockholm Inpatient Register to detect chronic disorders because disease information taken from the other sources was not available for all assessments [24]. In the analyses, multimorbidity was operationalised as “no disease”, “one disease”, and “two or more diseases”, because only a few persons had three or more diseases present at baseline (n = 22: 12 persons with and 10 persons without incident dementia).

#### Primary outcome measure

We were primarily interested in dementia progression in a broad, clinical sense. Therefore, as the primary outcome measures we modelled the change over time in the two major dimensions of functioning – cognitive and daily functioning – in the analyses. Global cognitive functioning was studied using MiniMental State Examination and functioning in Activities of Daily Living (ADLs) were measured using Katz ADL items [20], [25].

#### Statistical analysis

Characteristics as present at inclusion were presented using means and proportions – as appropriate – for the participants grouped by dementia diagnosis. Individual growth modelling was used to study the progression of cognitive and ADL functioning over time and to study the influence of dementia, multimorbidity, and their interaction on the progression of the outcome [26]. Time was treated as time (years) since the last KP assessment before dementia diagnosis for participants whom developed dementia and time since the KP assessment that was randomly chosen as the starting point of the follow up trajectory for participants without dementia. For each of the two outcome measures ADL score and MMSE, we first identified which unconditional individual growth model fitted the data best: linear growth or curvilinear growth. The aim of the first step was to find the models that best explained the within person change over time in each of the outcome measures. As such, the trajectory of each participant over time was characterized by an intercept and one or more slope parameters. These parameters are called the individual growth factors. Next, several predictors (dementia, age, sex, education, multimorbidity) were added one-by-one to assess whether they significantly (alpha <0.05) explained the (between person) variance in the individual growth factors. Age (years) and multimorbidity (0, 1, or 2 or more diseases) were taken into account in the models as age and multimorbidity at the last KP assessment before dementia diagnosis for participant whom developed dementia and at the KP assessment that was randomly chosen as the starting point of the follow up trajectory for participants without dementia. In the final models, dementia diagnosis, multimorbidity and their interaction was modelled to assess whether the influence of multimorbidity on cognitive and ADL functioning progression was specifically related to persons suffering of dementia or was also present in non-demented persons. These analyses were done unadjusted as well as adjusted for age, sex, education and living situation.

## Results

**Figure 1:**
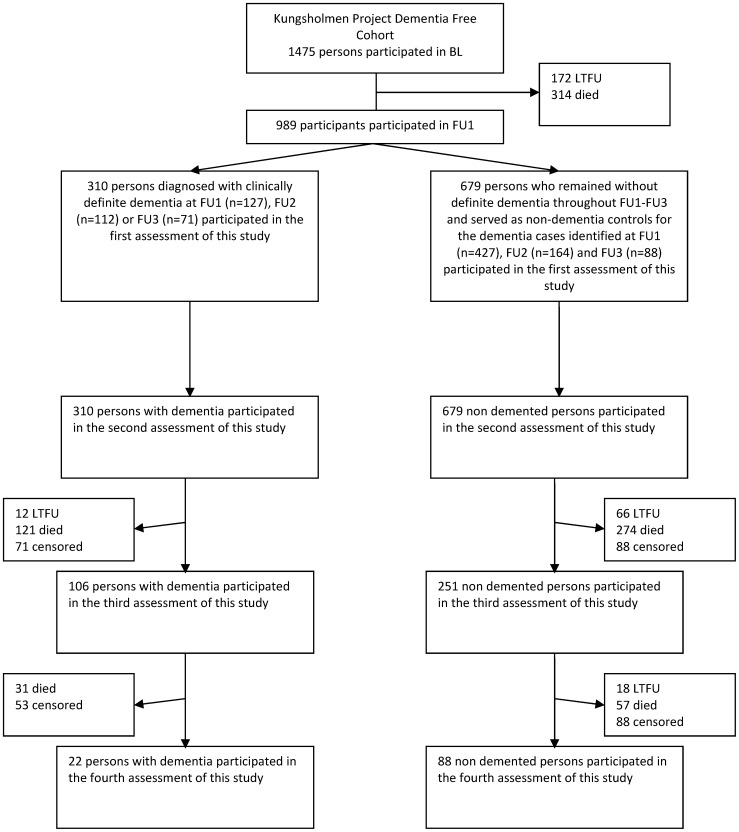
Study flow chart*. * BL, FU1, FU2, and FU3 indicate the original baseline and consecutive follow up assessments of the Kungsholmen Project. LTFU = lost to follow up because the participant withdrew from the study or could not be contacted. Died = lost to follow up because the participant died. Censored = the participant reached the final Kungsholmen Project Follow up assessment used in this study.

We followed 989 persons (310 persons with incident dementia and 679 persons who served as non-demented controls) longitudinally for a mean (SD) follow up period of 4.7 (2.3) years (figure 1). The participants had a mean (SD) age of 83 (4.7) at inclusion into this study and were in majority female (n = 759, 76.7%). Compared to non-demented persons, at inclusion in this study, dementia patients were older, more often female, had lower MMSE scores, had more ADL disabilities, and more often suffered from multimorbidity (Table 1). The dementia was in 251 (81% of 310) of the participants of Alzheimer type and in 59 (19% of 310) of non-Alzheimer type. Among the whole study group, 213 (22% of 989) participants had at least one or more diseases at the moment of inclusion in the current study (table 2). Among the 146 participants with one disease, most prevalent were chronic obstructive pulmonary disease (20, 14%), diabetes (17, 12%), osteo arthritis (17, 12%) and heart failure (15, 10%). Among the 67 participants with two or more diseases, heart failure (29, 43%), ischemic heart disease (17, 25%), chronic obstructive pulmonary disease (16, 24%) and diabetes (15, 22%) were most prevalent.

**Table 1:** Sample characteristics at baseline*.

		Non-demented persons (n = 679)	Persons with incident dementia (n = 310)	p-value for difference between groups
Age (years); mean (SD)		83 (4.7)	85 (4.5)	<0.001
Female sex; n (%)		501 (73.8)	258 (83.2)	0.001
Low education (≤7 years of formal education); n (%)†		315 (46.6)	174 (56.3)	0.005
Institutional living; n (%)		7 (1.0)	12 (3.9)	0.003
MMSE; mean (SD)‡		27 (2.1)	25 (3.3)	<0.001
ADL; n (%)	No disability	443 (66.3)	187 (60.5)	0.04
	Disability in 1 function	167 (25.0)	82 (26.5)	
	Disability in 2 functions	39 (5.8)	20 (6.5)	
	Disability in 3+ functions	19 (2.8)	20 (6.5)	
ADL score; mean (SD)§		0.8 (1.4)	1.2 (1.9)	<0.001
Morbidity; n (%)	No chronic diseases(other than dementia)	551 (81.1)	225 (72.6)	<0.001
	One chronic disease(other than dementia)	96 (14.1)	50 (16.1)	
	Two or more chronic diseases(other than dementia)	32 (4.7)	35 (11.3)	
N^o^ of morbidities; mean (SD)		0.3 (0.6)	0.4 (0.9)	<0.001
Follow up time (years); mean (SD)||		4.8 (2.4)	4.4 (2.0)	0.009

Characteristics of the sample at the moment of entry in this study, unless otherwise stated.

Education as was established at the original baseline assessment of the Kungsholmen Project.

Range 0–30, where a higher score indicated better functioning.

Range 0–12, where a lower score indicated better functioning.

^||^ Follow up time since the follow up assessment at entry in this study.

**Table 2:** Occurrence of Separate Conditions among Participants with one or more Diseases (n = 213) at Inclusion in this Study.

Condition, n (%)	Participants with one disease, n = 146	Participants with two or more diseases, n = 67
Hip fracture	14 (1)	8 (12)
Osteo arthritis	17 (12)	9 (13)
Rheumatoid arthritis	3 (2)	2 (3)
Atrial fibrillation	4 (3)	11 (16)
Cardiomyopathy	1 (1)	
Chronic rheumatic heart disease	1 (1)	
Heart failure	15 (10)	29 (43)
Hypertension	1(1)	6 (9)
Ischemic heart disease	9 (6)	17 (25)
Cerebrovascular disease	8 (5)	11 (16)
Diabetes	17 (12)	15 (22)
Disorder of thyroid		1 (1)
Cholelithiasis	1 (1)	2 (3)
Diverticula of intestine		2 (3)
Functional digestive disorder	1 (1)	3 (4)
Anemia	4 (3)	5 (7)
Chronic obstructive pulmonary disease	20 (14)	16 (24)
Cancer	15 (1)	7 (10)
Deafness	1 (1)	2 (3)
Disorders of the eye	8 (5)	2 (3)
Epilepsy	1 (1)	1 (1)
Parkinson disease		5 (7)
Peripheral nerve system		1 (1)
Alcohol dependence syndrome		1 (1)
Depression	4 (3)	6 (9)
Schizophrenia		1 (1)
Calculus of kidney and ureter		1 (1)
Hyperplasia of prostate	1 (1)	2 (3)

When we modelled the within person change over time in cognitive and daily functioning, the best fitting models contained both a linear and a quadratic individual growth factor (daily functioning: table 3, model 1; cognitive functioning: table 4, model 4), a random intercept (the variance component called “in initial status” in table 3 and 4), and a random slope (the variance component called “in linear rate of change” in table 3 and 4). A random quadratic slope was tested but did not further improve the fit of the unconditional growth models and was therefore constrained to zero by removal from the models [26].

**Table 3:** The Results of Fitting Different Individual Growth Models in Functioning in Activities of Daily Living*.

	Model 1: Unconditional individual growth model		Model 2: Effect of dementia, morbidity and their interaction, unadjusted		Model 3: Effect of dementia, morbidity and their interaction, adjusted†	
Parameter	Estimate	p-value	Estimate	p-value	Estimate	p-value
**FIXED EFFECTS**						
*Intercept* ‡	0.89	<0.001	0.69	<0.001	0.44	0.51
Dementia			0.16	0.21	0.05	0.66
Multimorbidity (0, 1, 2+)			0.37	0.003	0.29	0.01
Dementia*multimorbidity			0.35	0.06	0.28	0.11
*Linear rate of change* §	0.07	0.06	−0.06	0.15	0.02	0.82
Dementia			0.14	0.10	0.07	0.42
Multimorbidity (0, 1, 2+)			0.04	0.63	0.01	0.89
Dementia*multimorbidity			0.31	0.01	0.34	0.006
*Quadratic rate of change* ||	0.03	<0.001	0.01	0.04	0.02	0.21
Dementia			0.10	<0.001	0.10	<0.001
Multimorbidity (0, 1, 2+)			0.002	0.89	0.003	0.79
Dementia*multimorbidity			−0.04	0.03	−0.04	0.03
**RANDOM EFFECTS: VARIANCE COMPONENTS**						
In initial status	0.83	<0.001	0.79	<0.001	0.43	0.005
In linear rate of change	0.24	<0.001	0.11	<0.001	0.17	<0.001
Covariance	0.22	<0.001	0.17	<0.001	0.10	<0.001
Within person (residual)	2.10	<0.001	2.05	<0.001	1.98	<0.001

Functioning in Activities of Daily Living (ADL) was assessed with the six Katz ADL items scored on a three point scale (0 = no help needed, 1 = some help needed, 2 = much help needed), resulting in a score ranging 0–12, where higher scores indicate more help needed.

Adjusted for age, sex, education and living situation at the first assessment with which this participant was included in this study. For participants who developed dementia this was their status at the last KP assessment before dementia was diagnosed and for participants who remained without dementia the KP assessment that was randomly chosen as the starting point of the follow up trajectory for this study.

For model 2 and 3 this row indicates the initial status for participants without dementia or morbidity.

For model 2 and 3 this row indicated the linear rate of change for participants without dementia or morbidity.

^||^ For model 2 and 3 this row indicated the quadratic rate of change for participants without dementia or morbidity.

**Table 4:** The Results of Fitting Different Individual Growth Models in Cognitive Functioning*.

	Model 4: Unconditional individual growth model		Model 5: Effect of dementia, morbidity and their interaction, unadjusted		Model 6: Effect of dementia, morbidity and their interaction, adjusted†	
Parameter	Estimate	p-value	Estimate	p-value	Estimate	p-value
**FIXED EFFECTS**						
*Intercept* ‡	26.44	<0.0001	27.25	<0.001	27.73	<0.001
Dementia			−2.30	<0.001	−1.88	<0.001
Multimorbidity (0, 1, 2+)			−0.27	0.17	−0.04	0.83
Dementia*multimorbidity			−0.20	0.49	−0.20	0.47
*Linear rate of change* §	−0.78	<0.001	−0.18	0.004	−0.17	0.16
Dementia			−1.97	<0.001	−1.91	<0.001
Multimorbidity (0, 1, 2+)			0.01	0.92	0.02	0.84
Dementia*multimorbidity			−0.19	0.35	−0.27	0.17
*Quadratic rate of change* ||	−0.02	0.01	−0.02	0.05	−0.05	0.02
Dementia			0.02	0.27	0.02	0.38
Multimorbidity (0, 1, 2+)			−0.01	0.54	−0.01	0.46
Dementia*multimorbidity			0.04	0.18	0.06	0.09
**RANDOM EFFECTS: VARIANCE COMPONENTS**						
In initial status	3.81	<0.001	2.29	<0.001	1.41	<0.001
In linear rate of change	1.15	<0.001	0.31	<0.001	0.29	<0.001
Covariance	1.551	<0.001	0.51	<0.001	0.54	<0.001
Within person (residual)	4.26	<0.001	4.64	<0.001	4.54	<0.001

Cognitive functioning was assessed with MiniMental State Examination (MMSE), score ranging 0–30, where lower scores indicate worse functioning.

Adjusted for age, sex, education and living situation at the first assessment with which this participant was included in this study. For participants who developed dementia this was their status at the last KP assessment before dementia was diagnosed and for participants who remained without dementia the KP assessment that was randomly chosen as the starting point of the follow up trajectory for this study.

For model 2 and 3 this row indicates the initial status for participants without dementia or morbidity.

For model 2 and 3 this row indicated the linear rate of change for participants without dementia or morbidity.

^||^ For model 2 and 3 this row indicated the quadratic rate of change for participants without dementia or morbidity.

The associations of dementia, multimorbidity and their interaction with the outcomes Katz ADL and MMSE were first examined without adjustment (models 2 and 5, tables 3 and 4 respectively) and successively after adjustment for age, sex, education and living situation (models 3 and 6, tables 3 and 4 respectively). Dementia patients had a decline in daily functioning that continues to grow steeper with time, compared to participants not having dementia, as can be seen from the quadratic decline rate in ADL estimated as 0.10 (p<0.001) higher per squared year (year*year) since dementia onset in participants who developed dementia. At early stage follow up, if a dementia patient had one more comorbidity, this was associated with an additional 0.34 points per year (p = 0.006) in the linear ADL rate of change (model 3, table 3). However, as indicated by a quadratic fixed factor dementia*multimorbidity with a point estimate of −0.04 (p = 0.03, model 3, table 3), the quadratic decline rate in people with dementia and one more disease is lower as compared to participants with dementia with less disease. Persons without dementia had a worse baseline ADL score if they have more diseases, but their daily functioning over time was not significantly affected by multimorbidity.

**Figure 2:**
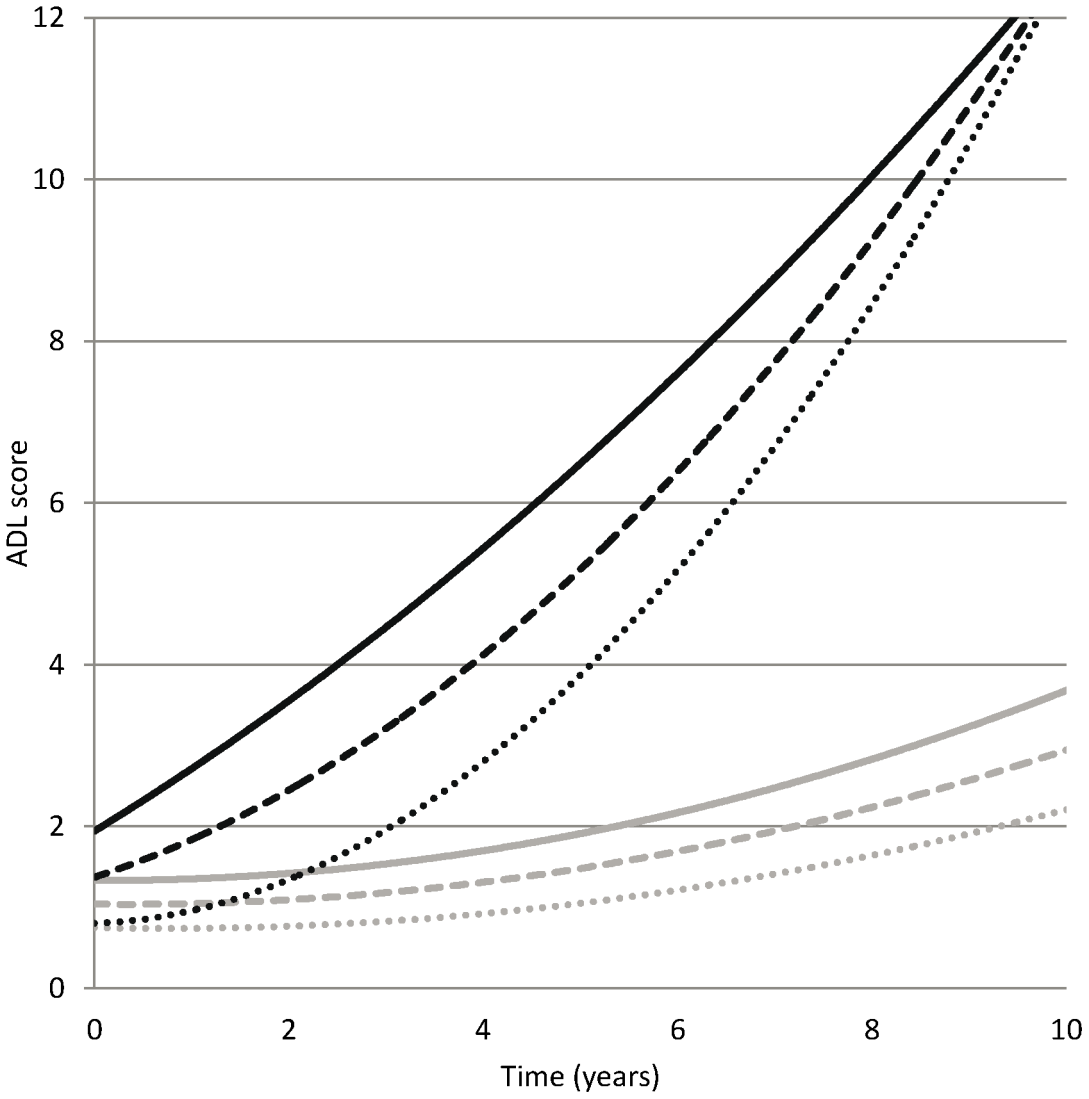
Mean Growth Curves for ADL Functioning. Mean Growth Curves for ADL Functioning* (Higher Score Indicates Worse Functioning) for Persons with Incident Dementia with 0 (black, dotted), 1 (black, dashed) or 2+ (black, solid) Diseases and Persons without Dementia with 0 (grey, dotted), 1 (grey, dashed) or 2+ (grey, solid) Diseases as Predicted by a Model 3 in Table 3. * Functioning in Activities of Daily Living (ADL) was assessed with the six Katz ADL items scored on a three point scale (0 = no help needed, 1 = some help needed, 2 = much help needed), resulting in a score ranging 0–12, where higher scores indicate more help needed.

To summarize how all the effects added up, the mean growth curves predicted by model 3 for a non-demented person with 0, 1 or 2 or more diseases and for a dementia patient with 0, 1 or 2 or more diseases were displayed graphically in figure 2. As explained above, whereas slopes for having 0, 1 or 2 or more diseases did not differ significantly for a non-demented person, the corresponding slopes did significantly differ for a dementia person. Further, whereas the intercepts differed significantly for having 0, 1 or 2 or more diseases, they did not differ significantly more when the person had dementia. We can observe that – irrespective of comorbidity – for participants with dementia the ADL decline rates were higher in the late period of follow up than those in early follow up. In addition, we can observe that at early follow up among dementia patients ADL decline rates were highest for people with two or more diseases, whereas at late follow up ADL decline rates were highest for dementia patients without diseases. At the end of follow up, ADL disability levels were comparable for all dementia patients.

**Figure 3:**
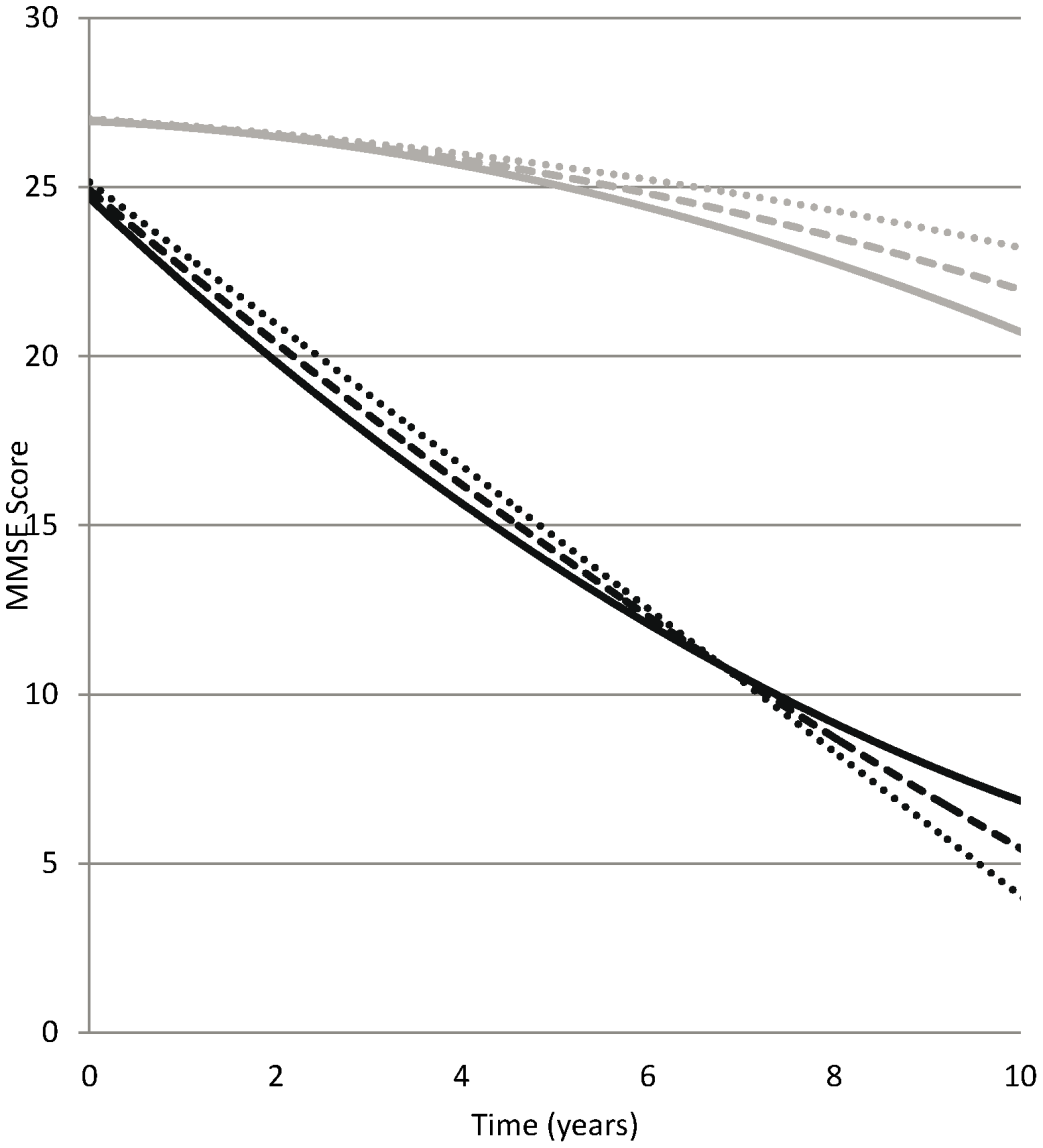
Mean Growth Curves for Cognitive Functioning. Mean Growth Curves for Cognitive Functioning* (Higher Score Indicates Better Functioning) for Persons with Incident Dementia with 0 (black, dotted), 1 (black, dashed) or 2+ (black, solid) Diseases and Persons without Dementia with 0 (grey, dotted), 1 (grey, dashed) or 2+ (grey, solid) Diseases as Predicted by a Model 6 in Table 4. *Cognitive functioning was assessed with MiniMental State Examination (MMSE), score ranging 0–30, where lower scores indicate worse functioning.

Participants with as well as without dementia showed a significant quadratic decline in cognition as indicated by a rate of −0.05 (p = 0.02) for participants without dementia (table 4, model 6 and figure 3). However, dementia patients had a significantly lower cognitive function already at baseline (−1.88, p<0.001) and – as expected – a steeper decline over time with an estimated increase in linear change rate in dementia patients of −1.91 (p<0.001). The effects of the number of diseases on cognitive functioning over time in participants with dementia were in the same direction as for daily functioning, but were much smaller and non significant.

## Discussion

This study showed that baseline chronic multimorbidity was significantly associated to accelerated decline in daily functioning but not in cognition in dementia patients. Whereas this effect was present in persons suffering from dementia, no effect could be identified in non-demented persons. Due to the combination of lower functioning in ADLs at baseline and faster decline, dementia patients with multimorbidity were about one to two years ahead of the decline of dementia patients without any co-morbidity. Remarkably, in persons without dementia, multimorbidity was not associated to hastened decline in ADLs, but was only associated with baseline ADL funtioning. We could not show convincingly any effects of the presence of additional diseases on cognitive function measured with MMSE. These findings have potential implications for clinical practice, as they stress the relevance of optimal treatment of co-morbidities in dementia patients.

These results are also relevant for our understanding of the pathophysiology of late-life dementia, because they suggest – as has been done earlier [2]–[4] – that physical factors may be involved in the clinical presentation of dementia. This is in line with the model outlined by Fotuhi et al and other researchers who suggested a dynamic polygon hypothesis [27], [28]. The dynamic polygon hypothesis provides a framework for thinking about aging and dementia that departs from the linear model proposed by the amyloid cascade hypothesis: it considers several pathological processes (e.g. amyloid aggregation, vascular damage) interlinked with positive or negative consequences of environmental exposures (e.g. exercise, obesity) to be affecting the size and functioning of the brain [28]. Multimorbidity may very well be one of the factors that add to the dynamic polygon ultimately resulting in the dementia phenotype. It is remarkable that multimorbidity did not affect change over time in ADLs when dementia was absent. These findings imply a strong interconnection between physical and mental health, where the greatest disablement occurs when both somatic and mental disorders are present.

When we compare these results with those of the study done by MacDonald et al which used Kungsholmen Project data on disease trajectories in more advanced stages of cognitive decline and in which multimorbidity did not affect the rate of progression [11], we may conclude that it is typically in the earliest phase of dementia onset that multimorbidity is of influence. Also, their study only looked at cognitive function measured with MMSE. In our as well as in another recent longitudinal population based cohort of persons with dementia baseline disease burden was not shown to be related to MMSE decline [13]. On the other hand, in a clinical cohort of dementia patients, disease burden was associated with MMSE decline [12]. In that study, comorbidity also tended to associate with an increased ADL functional decline, but without the effects being statistically significant. Further study is needed, both to better understand whether, how and when multimorbidity is part of the complex pathophysiology of dementia and how it is related to dementia progression, as well as to see whether optimizing the treatment of co-morbidities will result in a slower dementia progression. These studies could first seek to replicate our results in other observational studies with an additional focus on whether multimorbidity differently affects different aspects of disease progression and acts differently in different dementia subtypes. Also the studies could focus on whether there are discrete (clusters of) diseases that influence dementia disease progression or whether the effect of multimorbidity is independent of the diseases present.

Using data from Kungsholmen Project, we were able to study our research question in a sample of incident dementia cases taken from a general population who were systematically assessed for the presence or absence of dementia at several points in time and were followed over a long period of time of almost five years on average. This increased the external validity of these results, in comparison to for example clinical samples of dementia patients where the sampling is more prone to selective recruitment. Using KP data, we were also able to compare the effect of disease burden on cognitive and ADL decline in dementia patients with its effect in non-dementia controls. This is important, because in this way we could show that the faster decline in ADLs in dementia patients with multimorbidity was not independently associated with multimorbidity alone.

Despite the strengths of performing this study using data from a large population-based longitudinal cohort study such as KP, the study design also came with some limitations. The current study operationalized multimorbidity as the time-invariant number of diseases present at time is zero (KP assessment before time frame in which dementia became apparent for participants who developed incident dementia or a randomly chosen KP assessment for those participants not developing dementia). A time-varying multimorbidity status may be a better measure because it can better capture a study subject's true status. The same may be true for measures that do not only capture the number but also the severity of the conditions present, such as the Cumulative Illness Rating Scale-Geriatrics [29]. We were only able to use disease information taken from hospital admissions to establish the number of diseases present at baseline. This resulted in a smaller portion of people with multimorbidity than generally found. However, multimorbidity prevalence is known to be highly variable and dependent on the way it is determined [30]. This entails a general limitation of the external validity of studies into multimorbidity and also the results of our study cannot be immediately generalized to settings where multimorbidity was operationalized differently. By using only hospital admission diagnoses we counted the presence of multimorbidity that was less often present but probably also more severe. Unfortunately, the Kungsholmen Project does not provide sufficient information on the treatment the participants received for their chronic conditions to allow study into whether they were optimally treated and how this related to dementia progression. Participants of the Kungsholmen Project received usual care as it was provided by the Swedish healthcare system in the period 1987 to 1998.

Also important was that KP provided relatively few FU assessments that were also performed with relatively long time periods in between (approximately every three years). Further studies – such as the one from Solomon et al. [12] – using clinical samples taken, for example, from memory clinics (despite the results being more easily distorted by methodological problems such as selection bias) may complement population-based follow up studies, since in these clinical samples it is more feasible to have frequent FU assessments. Clinical samples may also offer more timely and more detailed description of dementia subtypes, than KP was able to provide. Nosological classifications of dementia syndromes have shown to change, and this is specifically difficult to handle in long running cohorts such as KP. Therefore, in the current study we refrained from studying how the effect of multimorbidity is different for different nosological subtypes and studied participants with a dementia as one group: the criteria for a dementia syndrome have remained fairly stable over the years. The long period over which we followed participants also meant a challenge to measuring cognitive functioning. MMSE is a measure that is fairly insensitive to small and subtle cognitive changes and certainly does not reflect the complexity of cognitive functioning. Measures that capture the complexity and subtleties of cognitive functioning better than MMSE were available in the Kungholmen Project, but not for all time points and the complete cohort. On other hand, empirical studies in dementia patients have also shown that over longer follow ups – as we had in our study – MMSE does capture cognitive change [31] and MMSE has been used previously for studying heterogeneity in cognitive trajectories in persons with dementia [12], [13].Selective attrition due to health and survival has been observed in previous longitudinal analyses of cognition [32] and may also have affected our results. To better understand the influence of follow status, as sensitivity analyses we reran the analyses presented in model 2 (table 3) and model 3 (table 4) respectively, now taking into account the statistical interaction of all fixed effects presented with loss to follow up due to death. Although this cannot definitely rule out that selective attrition resulted in biased estimates, the estimates for the fixed effects were comparable in the sensitivity analyses.

Next, the difficulty with any disease with a gradual onset, is that it is difficult to exactly frame when the disease becomes incident. Close assessment of the individual progression trajectories showed that many dementia cases already experienced a considerable decline before the FU assessment at which the diagnosis was formalized. Since we did not want to miss this early part of the trajectories in our analyses, we decided to use the last FU assessment before the dementia became incident as our baseline. Despite this, dementia cases still had worse cognitive scores than non-dementia controls already at baseline. This was in line with previous observations that dementia patients begin to experience declines in cognitive performance years before a formal diagnosis is made [33].

Our findings indicate that multimorbidity was associated with considerably accelerated decline in daily function amongst persons with dementia and may very well be one the physical factors that add to the dementia phenotype. The greatest disablement seemed to occur when both mental and physical impairments were present. At early follow up, the rate of ADL decline was largest in dementia patients with multimorbidity, at late follow up, the rate of ADL decline may be largest in dementia patients without multimorbidity at dementia onset.

This is a result that deserves further exploration. If it can be shown that optimizing the treatment of co-morbidities in dementia can improve the early prognosis of dementia patients, multimorbidity may offer one of the “windows” for the prevention of complications of dementia.
